# Draft Genome Sequences of Enterohemorrhagic *Escherichia coli* O103:H2 Strains Isolated from Feces of Feedlot Cattle

**DOI:** 10.1128/genomeA.00094-17

**Published:** 2017-05-11

**Authors:** Lance W. Noll, Jay N. Worley, Xun Yang, Pragathi B. Shridhar, Jianfa Bai, Jianghong Meng, Doina Caragea, T. G. Nagaraja

**Affiliations:** a Department of Diagnostic Medicine/Pathobiology, Kansas State University, Manhattan, Kansas, USA; b Veterinary Diagnostic Laboratory, Kansas State University, Manhattan, Kansas, USA; c Department of Computing and Information Sciences, Kansas State University, Manhattan, Kansas, USA; d Department of Nutrition and Food Science, University of Maryland, College Park, Maryland, USA

## Abstract

The enterohemorrhagic pathotype represents a minor proportion of the *Escherichia coli* O103 strains shed in the feces of cattle. We report here the genome sequences of 43 strains of enterohemorrhagic *E. coli* (EHEC) O103:H2 isolated from feedlot cattle feces. The genomic analysis will provide information on the genetic diversity and virulence potential of bovine EHEC O103.

## GENOME ANNOUNCEMENT

Cattle serve as the primary reservoir of enterohemorrhagic *Escherichia coli* (EHEC). The organism is shed in the feces of cattle, which can contaminate the hide and then carcass at slaughter, creating the potential for foodborne illness in humans (1). *Escherichia coli* O103 has been identified as the second most prevalent EHEC, next to EHEC O157, shed in the feces of feedlot cattle (2). The Centers for Disease Control and Prevention ranks O103 as the third most common EHEC, next to O26 and O157, identified in laboratory-confirmed EHEC infections in the United States (3). Disease outcomes with an EHEC infection can range from mild to bloody diarrhea, to more serious cases of hemolytic-uremic syndrome, which can be fatal (4). Although all EHEC strains are positive for Shiga toxin (*stx*_1_ and/or *stx*_2_) and intimin (*eae*) genes, the combination of these and presence of other major virulence genes, including enterohemolysin (*ehxA*), vary among EHEC O103 strains isolated from bovine feces (5), which is a likely indicator of genetic variability in the EHEC O103 population. Söderlund et al. (6) published the genomes of five EHEC O103 strains isolated from feces of cattle in Sweden, which, to our knowledge, remain the only publicly available sequences to date.

We report here the draft whole-genome sequences of 43 strains of EHEC O103:H2 isolated from feces of feedlot cattle in the United States (5). Bacterial DNA from 1 ml of overnight culture was extracted from each strain using the DNeasy blood and tissue kit with the QIAcube robotic workstation. An Illumina MiSeq platform (Illumina, San Diego, CA) was used to sequence the genomes, and genome libraries were constructed using Nextera XT DNA library preparation kit and MiSeq reagent kit version 2 (500 cycles) (Illumina, Inc.). SPAdes version 3.6.0 (7) was used to perform *de novo* genome assembly. Genome characteristics, including strain identification (ID), genome size, and number of contigs per genome are summarized in Table 1.

**TABLE 1:** Characteristics of enterohemorrhagic *Escherichia coli* O103:H2 strains isolated from feces of feedlot cattle

Strain	Genome size (bp)	No. of contigs	Accession no.
UMDKSU-2013-3-80A	5,407,763	325	MVLM00000000
UMDKSU-2013-3-104A	5,521,875	336	MVLN00000000
UMDKSU-2013-3-118A	5,521,709	338	MVLO00000000
UMDKSU-2013-3-135A	5,322,471	190	MVLP00000000
UMDKSU-2013-3-141B	5,317,169	181	MVLQ00000000
UMDKSU-2013-3-144B	5,317,321	184	MVLR00000000
UMDKSU-2013-3-174C	5,471,724	329	MVLS00000000
UMDKSU-2013-3-304D	5,447,896	343	MVLT00000000
UMDKSU-2013-3-314B	5,422,729	340	MVLU00000000
UMDKSU-2013-3-409B	5,447,263	335	MVLV00000000
UMDKSU-2013-3-539F	5,461,227	345	MVLW00000000
UMDKSU-2013-3-552F	5,458,651	356	MVLX00000000
UMDKSU-2013-3-557A	5,426,605	349	MVLY00000000
UMDKSU-2014-5-7C	5,471,330	302	MVLZ00000000
UMDKSU-2014-5-10D	5,477,735	305	MVMA00000000
UMDKSU-2014-5-58F	5,382,132	263	MVMB00000000
UMDKSU-2014-5-82A	5,382,514	265	MVMC00000000
UMDKSU-2014-5-89A	5,406,687	223	MVMD00000000
UMDKSU-2014-5-94C	5,411,198	205	MVME00000000
UMDKSU-2014-5-138A	5,459,858	296	MVMF00000000
UMDKSU-2014-5-139D	5,455,865	285	MVMG00000000
UMDKSU-2014-5-140E	5,466,422	275	MVMH00000000
UMDKSU-2014-5-288B	5,376,259	256	MVMI00000000
UMDKSU-2014-5-330A	5,600,684	348	MVMJ00000000
UMDKSU-2014-5-332A	5,661,314	375	MVMK00000000
UMDKSU-2014-5-370H	5,489,124	318	MVML00000000
UMDKSU-2014-5-548J	5,394,871	309	MVMM00000000
UMDKSU-2014-5-610A	5,452,796	290	MVMN00000000
UMDKSU-2014-5-611A	5,454,210	291	MVMO00000000
UMDKSU-2014-5-614A	5,454,099	300	MVMP00000000
UMDKSU-2014-5-650B	5,456,179	288	MVMQ00000000
UMDKSU-2014-5-655A	5,455,502	294	MVMR00000000
UMDKSU-2014-5-841G	5,503,496	299	MVMS00000000
UMDKSU-2014-5-863D	5,508,036	303	MVMT00000000
UMDKSU-2014-5-888D	5,593,208	361	MVMU00000000
UMDKSU-2014-5-925A	5,590,465	371	MVMV00000000
UMDKSU-2014-5-933A	5,653,062	394	MVMW00000000
UMDKSU-2014-5-941B	5,595,900	398	MVNA00000000
UMDKSU-2014-5-965B	5,452,108	387	MVNB00000000
UMDKSU-2014-5-1253A	5,421,805	343	MVMX00000000
UMDKSU-2014-5-1451D	5,546,045	388	MVNC00000000
UMDKSU-2014-5-1565C	5,787,539	382	MVMY00000000
UMDKSU-2014-5-1838G	5,431,181	359	MVMZ00000000

The 43 strains deposited here will add to the limited publicly available genetic information on EHEC O103. These genomes will be a valuable resource for investigations into the genetic features of this major foodborne pathogen, including research into the genome evolution of EHEC O103 compared to other EHEC serogroups, and insights into specific factors allowing for adaptation to a bovine host. This work will also provide an opportunity to study the potential virulence risk of these EHEC O103 strains to humans.

### Accession number(s).

The whole-genome shotgun sequence has been deposited at DDBJ/ENA/GenBank under the accession numbers listed in Table 1.
